# Impact of predictors upon the reduction of lipid parameters in family medicine practice: Impacto de preditores da redução dos parâmetros de lipídios na prática da medicina de família

**DOI:** 10.1590/1516-3180.2014.00321501

**Published:** 2015-08-21

**Authors:** Yusuf Cetin Doganer, Kurt Angstman, James Rohrer, Stephen Merry

**Affiliations:** I MD. Research Fellow, Department of Family Medicine, Mayo Clinic, Rochester, MN, USA; II MD. Associate Professor, Department of Family Medicine, Mayo Clinic, Rochester, MN, USA.; III PhD. Professor, Department of Family Medicine, Mayo Clinic, Rochester, MN, USA.; IV MD, MPH. Assistant Professor, Department of Family Medicine, Mayo Clinic, Rochester, MN, USA.

**Keywords:** Dyslipidemias, Cholesterol LDL, Hydroxymethylglutaryl-CoA reductase inhibitors, Lipids, Family practice., Dislipidemias, LDL-Colesterol, Inibidores de hidroximetilglutaril-CoA redutases, Lipídeos, Medicina de Família e Comunidade.

## Abstract

**CONTEXT AND OBJECTIVE:**

To evaluate predictors of changes in lipid parameters consisting of LDL-C (low-density lipoprotein cholesterol), TC (total cholesterol) and non-HDL-C (non-high density lipoprotein cholesterol) among primary care patients.

**DESIGN AND SETTING:**

Retrospective study conducted on family medicine patients.

**METHODS:**

Demographic features and other clinically relevant information were abstracted from medical records. The primary outcome was the difference in LDL-C level from initial testing to the index test. Secondary outcomes were the changes in TC and non-HDL-C levels between two measurements.

**RESULTS:**

Three hundred and eleven participants were included in the final secondary analysis. Multiple linear regression revealed that male patients (β = 4.97, P = 0.040), diabetes (β = 9.75, P = 0.003) and higher LDL-C levels at baseline (β = 0.35, P < 0.001) were positively associated with LDL variance, whereas longer time period (β = -0.15, P = 0.045) and familial hypercholesterolemia history (β = -7.56, P = 0.033) were negatively associated. Male patients (β = 8.45, P = 0.002), DM (β = 9.26, P = 0.011), higher TC levels at baseline (β = 0.35, P < 0.001) and taking statins (β = 7.31, P = 0.023) were positively associated with TC variance, whilst longer time period (β = -0.183, P = 0.031) and familial hypercholesterolemia (β = -10.70, P = 0.008) were negatively associated.

**CONCLUSION:**

In the present study, patients who were male, on statin treatment, diagnosed with diabetes and had higher baseline lipid values were more likely associated with better lipid outcomes at future testing.

## RESUMO

**CONTEXTO E OBJETIVO:**

Avaliar preditores de alterações nos parâmetros lipídicos que consistem em LDL-C (colesterol de lipoproteína de baixa densidade), TC (colesterol total) e não HDL-C (não colesterol de lipoproteína de alta densidade) entre os pacientes de cuidados primários.

**TIPO DE ESTUDO E LOCAL:**

Estudo retrospectivo realizado em pacientes de medicina familiar.

**MÉTODOS:**

Aspectos demográficos e outras informações clinicamente relevantes foram extraídos dos prontuários médicos. O desfecho primário foi a diferença de nível de LDL-C entre os exames iniciais e o exame índice. Os desfechos secundários foram as mudanças dos níveis de TC e não HDL-C entre as duas medidas.

**RESULTADOS:**

Trezentos e onze participantes foram incluídos na análise secundária final. Regressão linear múltipla revelou que os pacientes do sexo masculino (β = 4,97, P = 0,040), diabetes (DM) (β = 9,75, P = 0,003) e níveis de LDL mais elevados no início do estudo (β = 0,35, P < 0,001) foram associados positivamente com variância LDL, enquanto longo período de tempo (β = -0,15, P = 0,045) e história hipercolesterolemia familiar (β = -7,56, P = 0,033) foram associados negativamente. Pacientes do sexo masculino (β = 8,45, P = 0,002), com DM (β = 9,26, P = 0,011), níveis elevados de CT na linha de base (β = 0,35, P < 0,001) e tomar estatinas (β = 7,31, P = 0,023) associaram-se positivamente com a variância TC, enquanto longo período de tempo (β = -0,183, P = 0,031), hipercolesterolemia familiar (β = -10,70, P = 0,008) foram associados negativamente.

**CONCLUSÕES:**

No presente estudo, os pacientes que eram do sexo masculino, em tratamento com estatinas, com diagnóstico de DM e que tinham valores lipídicos basais mais elevados foram mais provavelmente associados a melhores resultados de lipídios em testes futuros.

## INTRODUCTION

Cardiovascular diseases (CVDs) are the largest cause of mortality and give rise to a substantial burden of health disparities in both developing and developed countries.^1^ Prevention and management of CVDs continue to be a major public health issue worldwide, with well-known risk factors.^2^ Patient outcomes may be improved if factors such as dyslipidemia, smoking or a sedentary lifestyle are controlled through comprehensive healthcare management. These approaches have been shown to have a positive impact through decreasing the mortality and morbidity rates linked with CVDs.^3^ Specifically, lipid-lowering therapies decelerate progression of atherosclerosis while decreasing recurrent cardiac events, as do a combination of intensive lifestyle and pharmacological interventions.^4^

The CVD prevention guidelines developed by the National Cholesterol Education Program expert panel (ATP III),^5^ which were updated in 2004,^6^ and the European Society of Cardiology (ESC) guidelines are primary resources for recommendations for lipid management in clinical practice.^7^ More recently, the American College of Cardiology/American Heart Association (ACC/AHA) task force published new recommendations, almost abandoning specific LDL targets.^8^ The recommendations of the ACC/AHA guidelines were extended to include all atherosclerotic CVDs (ASCVD), such as CHD and stroke, by calculating a risk assessment tool, specifically named the Pooled Cohort equations.^9^ ^10^ One consequence of this new US guideline is a lower risk assessment score for starting statin therapy, particularly for patients with no current cardiovascular symptoms.

Using the recommendations of the ACC/AHA guidelines as a basis for this study, LDL reduction was identified as a continuous outcome measurement instead of using dichotomous target outcomes for achievement. To our knowledge, few studies have investigated the association between patients' characteristics and variance of lipid parameters from baseline to the next measurement, as continuous variables in a randomized sample. 

## OBJECTIVE

We hypothesized that demographics (age, gender and smoking status) and possible clinical predictors such as body mass index (BMI) categories, familial hypercholesterolemia history, familial premature coronary heart disease (CHD), statin usage, comorbidities, interval periods between measurements, baseline low-density lipoprotein cholesterol (LDL-C), total cholesterol (TC) and non-high density lipoprotein cholesterol (non-HDL-C values), among primary care patients, would be associated with improvement of LDL-C, TC and non-HDL-C.

## METHODS

### Study sample and data

The present study was a secondary data analysis conducted on randomly selected adult primary care patients at a single multicenter outpatient practice in Rochester, Minnesota, USA. The lipid parameters of a random sample of patients for whom testing was ordered between March and September 2012 were electronically extracted (n = 400). The electronic medical records (EMRs) were retrospectively reviewed for baseline lipid results and dates, and the patients (n = 89; 22.3%) without prior lipid testing more than six years earlier were excluded from the study. The variables extracted included demographic characteristics (age, gender and smoking status) and clinical characteristics (BMI categories, familial hypercholesterolemia history, familial premature CHD, statin usage, comorbidities, interval periods between measurements, baseline LDL-C, TC and non-HDL-C values). Of the initial 400 patients, 311 (77.8%) were included in the final study cohort. The present study was reviewed and approved by our institutional review board.

### Outcome measurements

The primary outcome was the difference in the LDL level from baseline to the follow-up measurement. Secondary outcomes were changes in total cholesterol and non-HDL-C levels from baseline to the follow-up measurement. Follow-up serum lipid levels were subtracted from baseline lipid levels to detect a difference in lipid parameters. The time until the follow-up was based on the electronic medical records (EMRs) and was variable from patient to patient. Independent variables that were controlled for in the multiple linear regression model included age, gender, body mass index (BMI), smoking status, baseline lipid parameters (LDL-C, HDL-C, total cholesterol), statin usage and comorbid CVDs.

### Predictor variables

The personal characteristics and clinical features studied included age, gender (male versus female), BMI strata (< 30 kg/m2 versus ≥ 30 kg/m2), smoking status (current smoker versus former and non-smoker), time period passed between the two screenings (in months), use of a statin treatment (yes/no) and presence of any of the following diseases: hypertension, diabetes and CHD (include carotid artery disease, peripheral artery disease and past history of abdominal aortic aneurysm). Hypertension was described as: 1) use of hypertension medication; or 2) systolic blood pressure > 140 mmHg or diastolic blood pressure > 90 mmHg.^11^ Diabetes was defined as: 1) use of diabetes medication; or 2) fasting blood glucose greater than 126 mg/dl.^12^ Patients were accepted as either having or not having a diagnosis of CHD, according to their EMR documentation. Data on predictor variables were gathered just after the last lipid measurements, from the EMRs of patients admitted to family medical centers.

### Statistical analysis

Statistical analysis was carried out by using the SPSS 22.0 statistical package (SPSS Inc., Chicago, Illinois, USA). Descriptive statistics were computed for all independent variables in the study. Paired sample tests were performed to ascertain the relationship between baseline and follow-up differences in lipid parameters. The Kolmogorov-Smirnov test and visual inspection of Q-Q plots were used to determine whether the data presented normal distribution. Univariate associations were analyzed using the Spearman correlation. The Wilcoxon test was used to make comparisons on data that were not normally distributed. The Spearman correlation was used where indicated. Multiple linear regression analysis was used to examine the effects of predictors on lipid parameter change. P-values less than 0.05 were considered significant. 

## RESULTS

In our cohort of 311 adult primary care patients, the mean age was 55.3 ± 11.8 years (range: 22-75), and 53.1% were female. Almost half of the patients (54%) were obese, with a BMI of ≥ 30 kg/m2, and only 13.8% of the patients were smoking currently. The most frequent morbities were CHD (21.9%), diabetes (25.7%), hypertension (56.9) and non-coronary atherosclerosis (11.6%). The median length of time between the two measurements was 12 (1-72) months. The median length of follow-up was 12 months (range: 1-72), while the mean length of time between the two measurements was 17.64 ± 16.88 months. Almost half of the individuals (47.6%) were under treatment with a lipid-lowering agent. Other characteristics of the patients and mean cholesterol values at baseline are presented in Table 1.

**Table 1:**
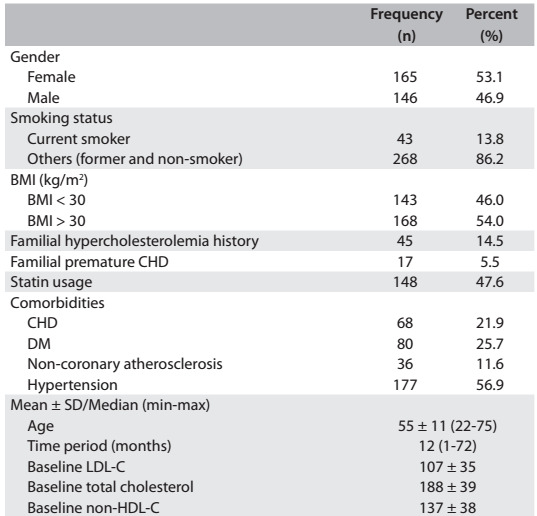
Descriptive statistics (n = 311)

BMI = body mass index; CHD = coronary heart disease; DM = diabetes mellitus; LDL-C = low-density lipoprotein cholesterol; non-HDL-C = non-high density lipoprotein cholesterol

**Figure 1:**
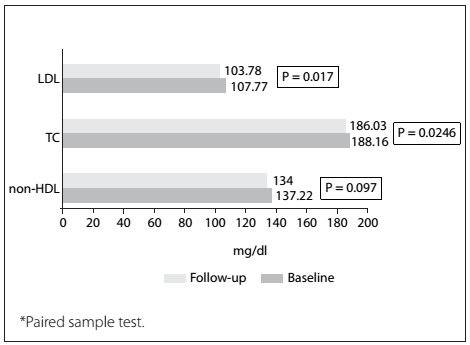
Reduction of overall lipid parameters from baseline to follow-up measurement (n = 311)*.

The mean lipid parameter values for LDL-C, TC and non-HDL-C at baseline were 107.8 ± 35.1 mg/dl, 188.2 ± 39.5 mg/dl and 137.2 ± 38.7 mg/dl, respectively. In addition, the mean values for LDL-C, TC and non-HDL-C at follow-up monitoring were 103.8 ± 33.2 mg/dl, 186.0 ± 37.8 mg/dl and 134.0 ± 36.6 mg/dl, respectively (P = 0.017, P = 0.246 and P = 0.097) (Figure 1). The change in LDL-C was highly correlated with TC and non-HDL-C alteration, regarding the metabolic affiliation to each other (r = 0.886, P < 0.001; r = 0.855, P < 0.001) (Table 2).

**Table 2:**
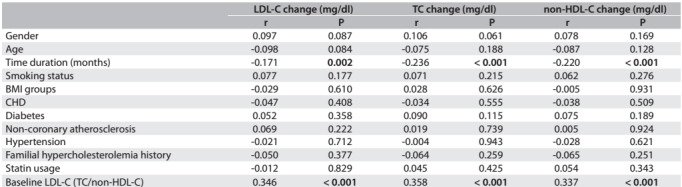
Spearman correlation (r) between changes of lipid parameters and descriptive characteristics (n = 311)

BMI = body mass index; CHD = coronary heart disease; DM = diabetes mellitus; LDL-C = low-density lipoprotein cholesterol; TC = total cholesterol; non-HDL-C = non-high density lipoprotein cholesterol

Multiple linear regression analysis on lipid parameter differences between baseline and follow-up measurements revealed a significant portion of the variance (LDL: adjusted R square = 0.250; analysis of variance, ANOVA, P < 0.001; TC: adjusted R square = 0.252; ANOVA P < 0.001; non-HDL-C: 0.237; ANOVA P < 0.001). Male patients (β = 4.97, P = 0.040), diabetes (β = 9.75, P = 0.003) and higher LDL levels at baseline (β = 0.35, P < 0.001) were positively associated with LDL variance, whereas longer time period (β = -0.15, P = 0.045) and familial hypercholesterolemia (β = -7.56, P = 0.033) were negatively associated. In other words, the changes seen at follow-up were the following: male patients, 4.9 points; patients with diagnosed diabetes, 9.7 points; and patients with higher baseline LDL-C values, 0.3 points. These patients achieved greatest improvement in LDL-C difference. Other demographic and clinical features were not significantly associated with LDL-C reduction (Table 3). Male patients (β = 8.45, P = 0.002), DM (β = 9.26, P = 0.011), higher TC levels at baseline (β = 0.35, P < 0.001) and taking statins (β = 7.31, P = 0.023) were positively associated with TC variance, whilst longer time period (β = -0.183, P = 0.031) and familial hypercholesterolemia (β = -10.70, P = 0.008) were negatively associated (Table 4). The predictors associated with non-HDL-C changes are shown in Table 5. 

**Table 3:**
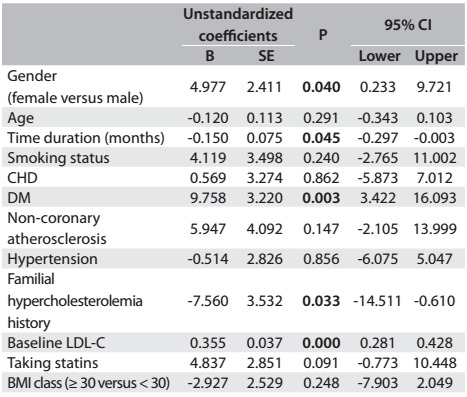
Multivariate linear regression analysis on predictors associated with the LDL-C change (n = 311)*

*R square = 0.279; adjusted R square = 0.250; ANOVA P value < 0.001. BMI = body mass index; CHD = coronary heart disease; DM = diabetes mellitus; LDL-C = low-density lipoprotein cholesterol

**Table 4:**
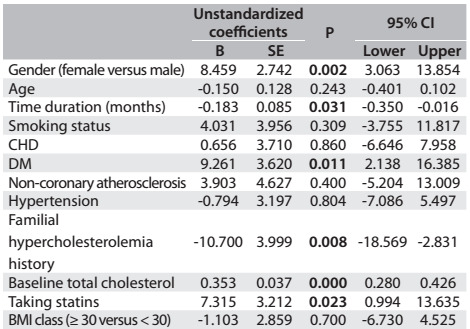
Multivariate linear regression analysis on predictors associated with the total cholesterol change (n = 311)*

*R square = 0.281; adjusted R square = 0.252; ANOVA P value < 0.001. BMI = body mass index; CHD = coronary heart disease; DM = diabetes mellitus

**Table 5:**
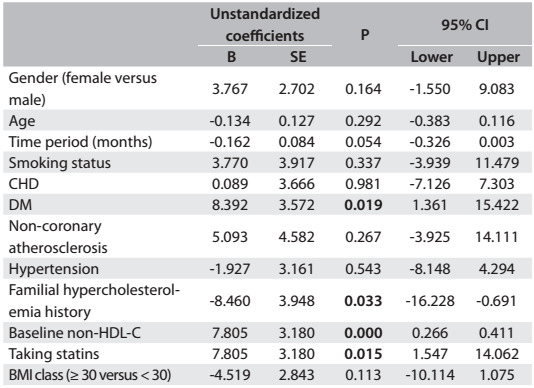
Multivariate linear regression analysis on predictors associated with the non-HDL-C change (n = 311)*

*R square = 0.267; adjusted R square = 0.237; ANOVA P value < 0.001. BMI = body mass index; CHD = coronary heart disease; DM = diabetes mellitus; non-HDL-C (non-high density lipoprotein cholesterol)

## DISCUSSION

In this sample of primary care patients, the serum level of LDL-C was significantly lower in the follow-up testing, compared with the serum levels of the baseline evaluation. In contrast with LDL-C, there were no significant reductions in serum levels of TC and non-HDL-C between baseline and follow-up measurements. Histories of diabetes, familial hypercholesterolemia and higher baseline levels were the common predictors affecting lower levels of all three lipid markers at follow-up. In addition, taking statins impacted TC and non-HDL-C variation, while being male and longer time duration between the samples were significant contributors to LDL-C and TC variation. In this study, we used the method of computing the reduction in lipid parameters as a continuous outcome, while many previous similar studies used a binomial outcome for determining whether lipid parameter goals were achieved. The Heart Protection Study (HPS)^13^ and the Cholesterol Treatment Trialists' (CTT)^14^ meta-analysis established that 1 mmol/l of LDL-C reduction was significantly associated with a 20% improvement in clinical cardiovascular events. Thus, the patient characteristics that predict improvement in cholesterol biomarkers could be important determinants. Since we were only able to find a limited number of similar studies with which to compare the findings of our present study, we had to make comparisons with previous studies focusing on whether lipid targets had been achieved.

Previous studies mostly focused on particular target control levels of lipid parameters set by different lipid guidelines for patients. These target levels may have been achieved through treatments with different types and dosages of statins.^15^ Contrary to these randomized, controlled clinical trials, the ACC/AHA recently declared that there would be no recommendations for specific LDL-C or non-HDL-C targets for primary and secondary prevention of atherosclerotic cardiovascular disease (ASCVD).^8^ Taking this point of view in our study, we used the method of calculating the reduction in lipid parameters as a continuous outcome, while previous similar studies used a binomial outcome of whether lipid parameter goals were achieved.

In an Asian study, goal attainment was determined to be directly associated with age and inversely related to baseline LDL-C.^16^ Before the new US guideline was issued, Kazerooni et al. stated that most of the observational studies conducted on LDL goal attainment disregarded classifying patients according to baseline LDL-C, in order to reduce heterogeneity in their baseline study samples. They suggested that LDL-C reduction from baseline levels should be used as an alternative method for evaluating improvement, instead of using LDL-C goals.^17^ Patients with higher LDL-C at baseline may have a relative advantage in achieving a larger change in LDL-C values, in relation to patients with lower initial LDL-C. In addition, physicians should consider selecting the initial drug and dose intensity of treatment based on the patients' baseline LDL-C levels, as well as on ASCVD risk assessments.

Dyslipidemia is very closely linked with insulin resistance, thus causing glycemic disorders. Based on this pathophysiological pathway, effective management of dyslipidemia plays a key role in preventing CVD, which is a crucial comorbidity in patients with DM.^18^ The current guidelines broaden the spectrum of statin usage, which is mainly indicated in patients with DM aged 40 to 75 years of age, as moderate-intensity statin usage to provide a decrease of 30% to 49% in LDL-C.^8^ Contradictory findings were determined in subgroup analyses on randomized control trials (RCTs) regarding cardiovascular risk reduction through statin therapy for diabetic patients, compared with all patients.^18^ In the LIPID study,^19^ the relative risk reduction was 24% in all patients and 19% in patients with DM, whereas 51% of all patients and 58% of diabetic patients had relative risk reduction in the GREACE study.^20^ In the present study, patients with DM displayed better improvement over time than patients without DM. This could potentially be due to enhanced therapeutic goals among diabetic patients, enhanced monitoring of diabetic patients and enhanced care treatment with care management, or a combination of factors.

The multicenter, multinational PROVE IT-TIMI 22 trial reported that both women and men profited from intensive statin therapy after acute coronary syndrome (ACS). The analysis on that study concluded that gender difference could not be a plausible reason in determining whether to implement intensive statin therapy.^21^ However, Victor et al. stated in their retrospective study that women with documented CHD were less likely to attain LDL-C and non-HDL-C goals.^22^ Confirming this finding, Rapeport et al. indicated that female patients were less likely to attain LDL-C goals than men, based on both the NCEP ATP III guidelines and the fourth Joint European Task Force (JETF) guidelines.^23^ We also found gender differences regarding LDL-C and TC variance. We detected that men made more progress than women regarding differences in LDL-C and TC. One possible explanation for our finding may be that women may have been less aggressively treated with lipid-lowering therapy, compared with men. Secondly, gender difference in lipid metabolism might lead to this type of variance.

Patients who were under statin treatment achieved greater improvement in TC and non-HDL-C. Statins not only lower LDL-C, but are also effective for TC and non-HDL-C, despite discrepancies regarding which marker is best for predicting possible CV events.^24^ ^25^ Although statin usage was not significantly associated with LDL-C variance in our sample population, TC and non-HDL-C improvement was noted. In addition, patients with high value as the start are likely to have decreased values at the retest, while patients with low values at baseline are likely to have increased values at the retest. This is due to some random variation in test scores. Extreme values may be due to chance rather than the actual disease.

Familial hypercholesterolemia (FH) is a common, but underdiagnosed and undertreated genetic cause of cardiovascular events, and it is linked with permanent elevated plasma LDL-C levels.^26^ However, once a heterozygote form of FH is diagnosed, it can be treated with statins or combined lipid-lowering therapies.^27^ According to our study findings, patients with a family history of FH were less likely to make progress in overall lipid parameter variations. Further detailed evaluation may be required among these patients due to inadequate improvement of variance, and more aggressive types of treatment could be applied.

### Limitation of the study

The present study has several limitations. We accept that our loss of information from 23% of the sample regarding the main outcome (lipid tests) was very high, and that this formed the significant weakness of the study. We assessed 311 patients because this was the number of participants available who fulfilled the requirements for secondary analysis using EMRs. 

The findings from this study possibly cannot be generalized to other patient groups given that the sample was randomly selected from one multi-site primary care practice group. We could not thoroughly observe the statin treatments regarding treatment options, durations, dosages or adherence. We were not able to ascertain when treatment for hyperlipidemia was started, and only noted when patients had follow-up data. In addition, we could not assess whether any therapeutic life-changing interventions occurred between two measurement dates in the electronic medical records.

## CONCLUSION

In the present study, patients who were male, were on statin treatment, presented type 2 DM and presented higher baseline lipid values were more likely to have better lipid outcomes at future testing. Longer durations between screenings and having a familial hypercholesterolemia history were less likely to be associated with achieving better lipid outcomes. There was no association between LDL-C change and statin usage in this retrospectively studied group, since some of the patients were being re-measured after stopping their statin treatment, some after starting statins, and some while on a maintenance dose of statins between the two lipid measurement points. Statin usage alone could not explain the differences in lipid parameters, and predictors affecting these variances require further studies with larger samples.
